# Telomere structure and telomerase in health and disease

**DOI:** 10.3892/ijo.2012.1611

**Published:** 2012-08-29

**Authors:** DANIEL E. GOMEZ, ROMINA G. ARMANDO, HERNÁN G. FARINA, PABLO LORENZANO MENNA, CAROLINA S. CERRUDO, P. DANIEL GHIRINGHELLI, DANIEL F. ALONSO

**Affiliations:** 1 Laboratories of Molecular Oncology, Department of Science and Technology and; 2 Genetic Engineering and Cellular and Molecular Biology, Department of Science and Technology, Quilmes National University, Buenos Aires, Argentina

**Keywords:** telomere, telomerase, tert, htr, dyskerin, cancer

## Abstract

Telomerase is the enzyme responsible for maintenance of the length of telomeres by addition of guanine-rich repetitive sequences. Telomerase activity is exhibited in gametes and stem and tumor cells. In human somatic cells, proliferation potential is strictly limited and senescence follows approximately 50–70 cell divisions. In most tumor cells, on the contrary, replication potential is unlimited. The key role in this process of the system of the telomere length maintenance with involvement of telomerase is still poorly studied. Undoubtedly, DNA polymerase is not capable of completely copying DNA at the very ends of chromosomes; therefore, approximately 50 nucleotides are lost during each cell cycle, which results in gradual telomere length shortening. Critically short telomeres cause senescence, following crisis and cell death. However, in tumor cells the system of telomere length maintenance is activated. Much work has been done regarding the complex telomere/telomerase as a unique target, highly specific in cancer cells. Telomeres have additional proteins that regulate the binding of telomerase. Telomerase, also associates with a number of proteins forming the sheltering complex having a central role in telomerase activity. This review focuses on the structure and function of the telomere/telomerase complex and its altered behavior leading to disease, mainly cancer. Although telomerase therapeutics are not approved yet for clinical use, we can assume that based on the promising *in vitro* and *in vivo* results and successful clinical trials, it can be predicted that telomerase therapeutics will be utilized soon in the combat against malignancies and degenerative diseases. The active search for modulators is justified, because the telomere/telomerase system is an extremely promising target offering possibilities to decrease or increase the viability of the cell for therapeutic purposes.

## Contents

1. Telomere structure
2. Telomeric binding proteins
3. Mechanisms of telomeric lengthening in mammals
4. Telomeres in cell immortalization and cancer
5. Other diseases associated with telomerase activity
6. Telomerase: structure and function
7. Other telomerase functions
8. Conclusion

## 1. Telomere structure

The ends of the eukaryotic chromosomes have special properties when compared with chromosomal ends created by stochastic breakdown. More than 70 years ago Muller and McClintock established that the ends of eukaryotic chromosomes had a different structure (1,2). Using X-rays in order to generate chromosomical rearrangements in *Drosophila*, Muller could not recover the chromosomes that were deleted in one end, in contrast with the chromosomes that have internal deletions or translocations. He concluded that this end should have a special structure needed for the integrity of the chromosome and he named it ‘telomere’. Few years after, McClintock demonstrated in maze that broken chromosomes could stick among them to form a dicentric chromosome highly instable by frequents breakdowns during mitosis, resulting in the loss of a part of the chromosome. With these experiments he concluded that one essential function of the telomeres was to protect the fusion of the extremes of the chromosomes.

Decades later, different molecular techniques revealed that the extremes of the chromosomes consisted in repetitions rich in guanidine (3).

The term telomere makes reference to a big nucleoprotein complex found in the extremes of the chromosomes, where their structure is different from the rest of the chromatin (4). Functional telomeres are stable structures not subjected to degradation, recombination or fusion with other chromosomal ends. Furthermore, they are not detected by the systems of damage recognition of DNA, although technically, a chromosomal end constitutes a cut in the double chain of DNA (5). These properties of the eukaryotic telomeres are identified usually as the ‘capping function’ exerted in the chromosomal ends (4). Telomeric DNA of virtually all the eukaryotic organisms consists in short and repetitive sequences (6). The primary sequence and the organization of such repetitions are clearly related in many species, since such repetitions usually contain groups of 3 or more G, and the strand that contains them (called string G enriched) always constitute the end 3′ of the chromosomes (6).

The number of repetitions in the telomere varies widely among different organisms and also among different telomeres in one organism. For all vertebrates, including humans, the repetitive sequence is d(TTAGGG) (7). Individual telomeres may contain only a few kb of repetitive sequence in the form of double chain, as in some transformed cell lines or even >100 kb, as in some mouse cells. Most of that DNA is organized in nucleosomes and only the more distal part could be found in a special conformation as non-nucleosomic chromatin that could be analogous to the special structure found in telomeres of yeast or *Tetrahymena*(8).

**Figure 1.**
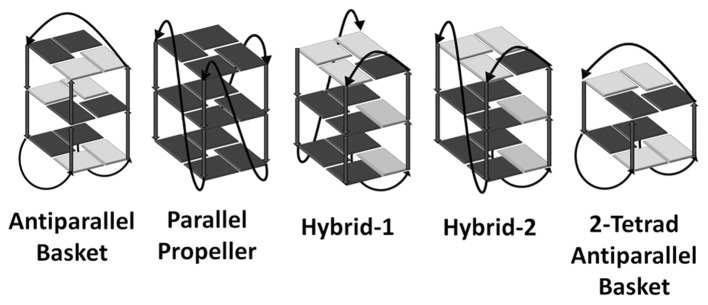
Schematic representation of G-quadruplexes, showing its different possible conformations.

**Figure 2.**
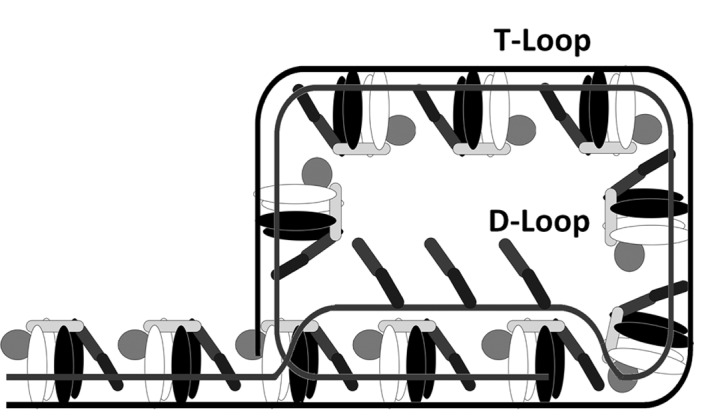
Schematic interpretation of a telomere in a T-loop configuration with a displacement D-loop.

The G-rich strand of telomeric DNA is always oriented 5′-3′ towards the terminal portion of the chromosome and had a protrudent extreme of ∼200 nucleotides (9) as consequence of the problem of terminal replication. The 3′ protruding G-rich strand can form complex structures of telomeres, the G-quadruplexes. G-quadruplexes, assume different conformations, clearly described by Hänsel *et al*(11). They are multi-stranded structures held together by square planes of four guanines (G-quartets) interacting by forming Hoogsteen hydrogen bonds (Fig. 1) (10,11). Formation of such structures on telomeres can be a problem for DNA replication and telomere elongation by telomerase. Telomeres form large loop structures called telomere loops, or T-loops which serve to sequester the chromosome terminus (Fig. 2). Here, the single-stranded DNA curls around in a long circle stabilized by telomere-binding proteins. At the very end of the T-loop, the single-stranded telomere DNA is held onto a region of double-stranded DNA by the telomere strand disrupting the double-helical DNA and base pairing to one of the two strands. This triple-stranded structure is called a displacement loop or D-loop (10) (Fig. 2).

## 2. Telomeric binding proteins

**Figure 3.**
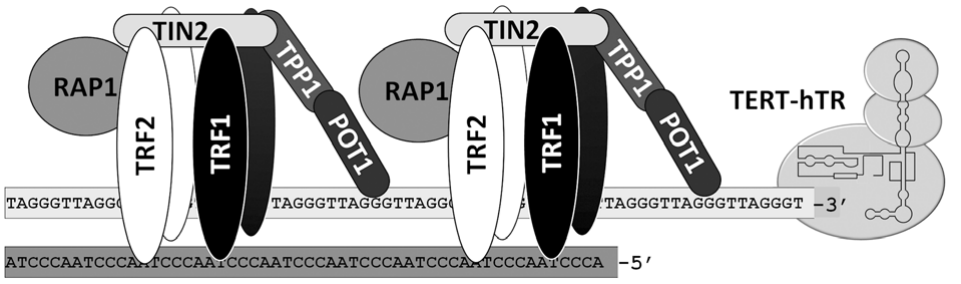
Schematic representation of telomeric binding proteins.

Fig. 3 shows different telomeric proteins that bind to mammal telomeres. One general concept relates to the evolution of telomere-associated proteins (12). Although telomere function is conserved in different organisms, the architecture and composition of telomere-associated proteins is remarkably varied and seems to have changed rapidly along evolution (13). In humans, telomeres are bound by a six-protein complex called shelterin, comprised of TRF1 and TRF2 which in turn recruits RAP1, TIN2, TPP1 and POT1 which interacts with ss and ds telomeric DNA (14). Shelterin prevents the activation of a DNA damage response (DDR) at chromosome ends and acts in the regulation of telomerase activity at chromosome ends (15).

In human cells, the best known telomeric binding proteins are TRF1 and TRF2. The first one to be identified was TRF1 (16). The C-terminal sequence of this protein, also named teloboxes, recognize specifically a telomeric DNA fragment, however, they require a dimerization in the N-terminal end to bind firmly to the DNA. TRF1 is a negative regulator of the telomeric length (17).

Another protein binding to the repeat TTAGGG is TRF2, which is also a negative regulator of telomeric length (18). TRF2 has unique functions in the telomere as stabilizer of the protruding G string and is able to prevent telomeric fusions (19). The overexpression of a dominant negative mutant of TRF2 may cause premature senescence (20), and the activation of the apoptotic cascade mediated by the protein kinase ATM and p53 (21). Their properties in the telomeric length and in terminal protection could be related to its location on the telomeric capping (22).

TRF1 and TRF2 do not allow telomerase activity, thereby restricting or inhibiting telomere elongation (19). Also, they are involved in two important pathways (ATM and ATR) which are potential sensors of DNA damage. ATM is a member of the phosphoinositide 3-kinase related protein kinase family (PIKK), along with ATR. These molecules have serine/threonine protein kinase activity with S/TQ consensus phosphorylation motifs on their specific substrates. ATM and ATR are involved in inducing cell cycle arrest upon DNA damage response pathway or through the activation of downstream kinases, Chk1 for ATM and Chk2 for ATR. The ATM and ATR signalling kinases are capable of inducing DNA damage response at telomeres in conditions where shelterin activity is impaired or reduced, for example due to telomerase shortening in senescent cells (23). ATM has a complex role at telomeres interacting with both TRF1 and TRF2 having an effect on telomere protection and TRF1-mediated telomere length regulation. On the other hand, ATR pathway is triggered in response to DNA replication stress, single stranded DNA replication stress and single stranded DNA accumulation. The genetic analysis of the repression of the DNA damage response in mouse cells has ascribed specific and separated functions for TRF2 and POT1 (24). To that effect POT1 inhibits ATR-mediated responses and TRF2 inhibits ATM.

TRF2 recruits in human telomeres the protein RAP1 whose overexpression causes telomeric elongation (25). Human Rap1 is encoded by the gene TERF2IP, localized in chromosome 16 with pseudogenes in chromosomes 5 and 22. Rap1 is a component of the shelterin complex at mammalian telomeres, but its *in vivo* role in telomere biology has remained unknown up to date. Recently, Martinez *et al* have shown that Rap1 deficiency is dispensable for telomere capping but leads to increased telomere recombination and fragility (26). Besides its role at telomeres, RAP1 also has extratelomeric roles. Rap1 is a transcription factor that controls the expression of glycolytic enzymes and ribosomal genes (27). RAP1 not only binds to telomeres but also to other non-telomeric sites preferentially throughout the recognition of TTAGGG consensus motif (26). Extratelomeric RAP1 binding sites are enriched at subtelomeric regions, highlighting a conserved role for RAP1 in subtelomeric silencing. In addition, RAP1 was shown to bind to non-coding regions in chromosomes 2, 11 and 17, which are enriched in TTAGGG tandem repeats, raising the possibility that RAP1 might prevent fragility and recombination at these genomic sites. RAP1 can interact with factors other than TRF2 to help gene transcriptional regulation, such as genes of the insulin secretion, peroxisome proliferator-activated receptor (PPAR), signaling, and growth hormone pathways. In another role beyond telomeres, RAP1 has been shown to play a role as an essential modulator of the nuclear factor-κβ (NF-κβ) mediated pathway (28).

In the case of TIN2, the gene that encodes this protein is TINF2, formed by 9 exons in a sequence of 3032 bp, giving origin to two transcripts, isoform 1 with 9 exons and an alternative with 6 exons. Their functional difference is unknown. Inhibition of PARsylation of TRF1, forms a tertiary complex with tankyrases regulating its activity (29).

Human TPP1 is a protein encoded in humans by the ADC gene in chromosome 16q22.1. It has 544 amino acids and a molecular weight of 57.7 kDa. Recently Tejera *et al* have demonstrated that this protein is needed for telomerase recruitment *in vivo*(30).

POT1 is an acronym for ‘protection of telomeres’. The gene of this protein localize in chromosome 7 in humans. It contains 22 exons, giving place to 5 variants by alternative splicing. Its higher level of expression is in testis, while the lower is found in muscle and colon. TPP1 binds to POT1 by its carboxyl end. This union is constant and it is needed for localization of POT1 in telomeres and also for the regulation of the telomeric length interacting with telomerase. In brief, POT1 regulates telomeric length acting as an activator or inhibitor of telomerase depending the position of POT1 in the 3′ overhang. It contributes to telomeric protection, inhibiting the formation of anafasic bridges and avoiding the action of the DNA repair system if it erroneously recognizes a 3′ overhang as damage (31).

Another important structural parameter governing telomere function is that they also contain RNA, called TERRAs (telomere repeat containing RNAs) (32). These RNAs are created from telomeres themselves, through transcription from CpG islands with promoter activity present in the subtelomeric regions (33). The transcription of TERRAs is believed to be mediated by TRF1, through an interaction with Pol II. It is unclear how TERRAs associate with telomeres. Functionally, TERRAs are implicated in the formation of telomeric heterochromatin, telomere protection and negative regulation of telomerase. In addition to their roles on the telomeric chromatin, TERRAs also exert competitive and non-competitive inhibition on telomerase itself: they can hybridize with hTR, the telomerase RNA, with high affinity and interact with the catalytic subunit hTERT.

The shelterin components are quantitatively associated with telomeres, require the TTAGGG repeats for assembly and are present at telomeres throughout the cell cycle. However, shelterin exerts its roles on telomere function through the transient recruitment of accessory factors (34) and can be viewed for some aspects of telomere function as an assembly line for them.

There are also TRF1 and TRF2 associated factors. The main factor associated to TRF1 is tankyrase. Tankyrase is a poly-ADP ribose onto target substrate and is particular to TRF1. The enzyme is a molecule with well defined catalytic domain (PARP), at the C-terminus, a His-Pro-Ser rich domain in the first N-terminal 181 residues and, at the middle of the molecule, 24 ankyrin-repeats involved in protein-protein interactions. The ankyrin repeats of tankyrase bind TRF1 through an interaction with its N-terminal acidic domain and add the poly-ADP-ribose chain to Glu residues (35). This specific posttranslational modification leads to a significant decrease in the affinity of TRF1 for the DNA, and results in the ubiquitinization and proteasomal degradation of the protein. Therefore, tankyrase is a positive regulator of telomere length through its action of effectively removing TRF1 from telomeres. Tankyrase has also other roles in the cytoplasm, where it associates with the Golgi and PARsylate substrates, in a process important for insulin-stimulated vesicle transport. In addition to telomere length regulation and the timely events necessary for the metaphase to anaphase transition, tankyrase 1 is also important for the protection of telomeres. Also, tankyrase 1 has a closely related homolog called tankyrase 2: the two molecules share a very high degree of homology in their domains and tankyrase 2 differs from tankyrase 1 in that it is lacking the N-terminal His-Pro-Ser rich domain (36). Tankyrase 2 was found to be present at telomeres, to have the capacity to PARsylate TRF1 and to induce telomere elongation as effectively as tankyrase 1.

PINX1 is a TRF1-associated telomerase inhibitor which associates with TRF1 through interaction with the F142 motif present in the TRFH domain, and therefore represents one of the activities able to bind this motif (37). The binding is highly specific as no detectable association was found with TRF2. The domain in PINX1 responsible for the interaction with TRF1 (termed TID for telomerase inhibitory domain) is present in the 75C-terminal residues. The presence of the TID domain on PINX1 is needed and sufficient for telomeric targeting through interactions with TRF1. PINX1 is able to simultaneously interact with the telomerase catalytic subunit providing the enzyme a physical link with TRF1 (38). PINX1 has been shown to mediate telomere length control through inhibition of telomerase. In cells, overexpression of PINX1 or of the TID domain leads to telomere shortening, demonstrating that this factor is a negative regulator of telomere length.

There are also TRF2-associated factors (Apollo, MRN complex, PNUTS, MCPH1, WRN/FEN1 and ORC complex TERRA). Apollo (39) is a well-characterized 5′-3′ exonuclease domain and a metallo-β-lactamese motif. Apollo is also involved in interstrand crosslink repair, besides it roles at telomeres (40). The recruitment of Apollo to telomeres is completely dependent on TRF2. The interaction between the two molecules occurs between the F120 of TRF2 and a TBM present at the C-terminus of Apollo (37). Apollo presumably contributes to the formation of the overhang on leading strand telomeres, and the effective replication through the telomeric tract (41).

Another associated factor is the MRN complex, composed of MRE11, RAD50 and NBS1, which has been implicated also in generating the overhang on the leading telomeric strand, similar to Apollo (42). However, a major difference is that, unlike Apollo, mutations in the components of the MRN complex do not show effects on the overhang on their own, but after induction of telomere dysfunction by, for instance, removal of TRF2. The MRN complex is implicated in the response to certain types of DNA damage, for instance double strand breaks, and is important in activating ATM and as an effector downstream of ATM (43). The MRN complex could also promote telomere elongation by downregulation of TRF1.

PNUTS, the protein phosphatase 1 nuclear-targeting subunits, binds to the catalytic subunit of PP1 and inhibits dephosphorylation of PP1 targets. PNUTS has a role in the general damage response (44).

MCPH1 has three BRCT domains, two of which, at the C-terminus, functioning as phosphopeptide binding modules on targets involved in the DNA damage response (45). Also MCPH1 is known to have a role in both the ATM and ATR pathways, in that it is important for recruitment of MDC1, NBS1 and RAD1 to sites of DNA damage.

WRN and FEN1 act as a pair and both bind to TRF2. Mutations in the WRN locus are associated with Werner syndrome, a progeroid syndrome characterized by premature aging, high incidence of diabetes and cardiovascular diseases, as well as a cellular phenotype of premature senescence, high chromosomal instability and telomere dysfunction (46). WRN is a member of the RecQ family of helicases. Unlike the other members of the family, WRN, in addition to DNA helicase activity has a 3′-5′ exonuclease domain found at the amino terminus. WRN has branch migration activity and works on different substrates, including telomeric DNA (47). WRN was found to co-operate functionally at telomeres with shelterin proteins TRF2 and POT1, and to be involved in telomere processing during S phase. A physical association between WRN and TRF2 has been documented both *in vivo* and *in vitro*(48). TRF2 and FEN1 have also been reported to interact. Both FEN1 and WRN are recruited to telomeres in S phase (49), consistent with an important role with replication, perhaps in reinitiation of stalled forks. These observations are compatible with the view that telomeres constitute a ‘fragile site’ on the chromosome, requiring specific activities to promote effective replication. Without the WRN helicase activity, and FEN1 ‘gap’ exonuclease activity, both required for efficient lagging of strand synthesis, the replication forks experience collapse leading to incomplete replication of telomeres (50).

The case of ORC complex-TERRA is a very interesting one. The origin recognition complex (ORC) was discovered in yeast as a six-subunit complex recognizing specifically the ARS chromosomal element essential for DNA replication (51). Importantly, TRF2 recruits the ORC complex at telomeres as well, and the ORC complex is important for telomere function. Functional studies were performed by siRNA of the ORC2 subunit, which resulted in chromosomal aberrations such as increased numbers of signal-free ends and double minute chromosomes, suggestive of strong replication defects. Telomere loss and an increase in t-circle formation were also observed, arguing for the ORC complex repressing recombination as well (52). Further the stability of the TRF-ORC1 complex was increased by TERRAs, the telomere-transcribed RNAs, as they can associate with both proteins and forms a ternary complex (53). Depletion of TERRA leads to a change in chromatin structure as detected by histone modifications (53). Thus, by inference, it appears that TERRA is important for the maintenance of heterochromatization of telomeres.

The complex ERCC1-XPF is a structure-specific endonuclease involved in nucleotide excision repair (NER) pathway and crosslink repair (54). A role for XPF-ERCC1 is suggested in processing the overhang at normal telomeres, affecting both strands resulting from leading or lagging strand DNA synthesis. At present, this complex is therefore implicated in overhang processing and, to some extent, telomere regulation.

The Ku complex is also part of this complex structure. Ku70/Ku80 is a heterodimer conserved along evolution. In human cells, the Ku complex is essential for viability through its role in repressing rapid deletions at telomeres. It is suggested that the presence of TRF2 at telomeres and its role in T-loop formation are important for masking the ends of chromosomes from Ku recognition.

In addition to anchor sites located within telomerase itself, it has been suggested that telomerase-associated proteins facilitate the repeat addition processivity of the enzyme *in vivo*. For example, the TPP1-Pot1 heterodimer stimulates the activity and processivity of human telomerase *in vitro*. TPP1 has been shown to interact with hTERT *in vitro* and mutation of a conserved glycine residue in the hTERT TEN domain was shown to suppress the stimulatory effect of TPP1-POT1 on human telomerase processivity *in vitro*(55). These results are important because they identify a physical link between the human shelterin complex and telomerase and provide new insight into the mechanism of processive telomere synthesis.

## 3. Mechanisms of telomeric lengthening in mammals

A fundamental telomeric function is to act as a kind of buffer to support the erosion of DNA chromosomic ends due to the problem of terminal replication. Conventional DNA polymerases are unidirectional and they cannot copy all bases in the 3′ end after primer removal. The result is that in each replication cycle, a given end of the chromosome cannot be synthesized completely and is lost. If organisms could not solve this replicative problem they could not pass they genetic charge completely from generation to generation. As a consequence, all the species should have, at least at the level of germinal cells, a mechanism that prevents the incomplete replication of the genomes.

Different organisms have acquired evolutively different methods to prevent the lost of DNA from the ends of their chromosomes; however, most of the mammals use telomerase, a specialized retrotranscriptase that will be described later.

Human cell lines that lack telomerase activity, are capable of maintaining or elongating their telomeres by alternative means, this has been called ALT (alternative lengthening of telomeres) (56). In mammals that exhibit ALT, sequences of DNA are copied from a telomere to the other suggesting that ALT would involve processes of homologous recombination (57).

In *Saccaromyces cerevisiae*, two ways of homologous recombination exist involved in ALT, which depend of RAD50 or RAD51 (58). Also, the helicases RecQ (WRN and BLM in mammals) are required for ALT in yeast (59). ALT mechanisms could be increased by the elimination of mismatch repair pathways (MMR) in yeast, and MMR machinery inhibits homologous recombination (60).

## 4. Telomeres in cell immortalization and cancer

Based on the initial experiments that demonstrated that in fibroblasts, in the absence of telomerase, telomeres shorten in direct relationship with the increasing of the number of cell divisions, it was suggested that the loss of telomeres could explain cell senescence after a given number of duplications *in vitro*(61).

Studies of fibroblasts cultures have indicated that the telomeric length would be better than the real age of the donant to predict the replicative capacity of their cells (62). It has been proposed that the loss of telomeric repeats, when reaching a critic telomeric length, induces a signal of damage to DNA that results in the exit of the cell cycle and replicative senescence (63). According to this model, telomeres act as a mitotic clock that determines the replicative life of a cell (64). The use of the telomeric length as a marker of the cells’ replicative history has been also described by Chang and Harley who demonstrated that telomeric length decreases in function of the passages of endothelial cells in culture (65). Bodnar *et al* gave the most definitive proof showing that the reintroduction of the catalytic compound of telomerase in primary human fibroblasts and in endothelial cells that lack telomerase activity elongated the telomeric repetitions in this cells resulting in a significant increase in their replicative life (66).

The ‘telomeric hypothesis of cell senescence and immortalization’ describes the potential relationship between telomeric dynamics and immortalization (61). Telomerase is expressed in cells of the germinal line which have long telomeres (∼10 kb) (67); in normal somatic cells, telomerase is repressed and telomeres shorten up to a critic length whereas the cells stop dividing (62).

The detention of the cell cycle that is imposed in these cells is maintained by signals that activate the pathways of the tumor suppressor genes p53 and Rb. This limit of the stage of mortality (M1) could be altered by the transformation with viral agents such as SV40 or the antigen T that inactivate p53 or Rb and permit to the cells to suffer other additional cell divisions. These cells can not yet divide indefinitely and are not considered immortal. This proliferation is followed by ulterior telomeric erosion, until the cells reach a second stage of mortality (M2) whereas the telomeres are critically short (∼3 kb) (68). In this stage of crisis, the cells could continue proliferating but showing high rates of apoptosis, triggered by important chromosomic aberrations, where there is no net increase in cell number. Progression beyond this point is a very rare event which requires the alteration by mutation of additional oncogenes and tumor suppressor genes. However, the cells that succeed in overcoming M2 correlate strongly with the reactivation of telomerase activity, the stabilization of telomeres and the acquisition of an immortal phenotype. Telomerase is active in the germ line as well as in stem cells but is inactive in most of the somatic cells. On the other hand, telomerase is active in most of immortalized cell lines and in 85–90% of human tumors. In a study carried out in culture cells, representing 18 different human tissues, it was found that 98 of the 100 studied cells, telomerase was positive but was negative in the 22 mortal populations analyzed. In the same way, telomerase activity was found in 90 of the 101 biopsies that represented 12 human tumor types, but none of the 50 normal somatic tissues was positive (69).

Clinical experience in cancer patients indicates that some primary cancers and most metastatic lesions undergo a period of dormancy before entering a stage of progressive growth. Although, this may be the most crucial step in cancer progression, the mechanisms underlying the conversion from a dormant to an actively growing state have not been elucidated. Few studies have focused on the role of telomerase on cancer dormancy. Gauthier *et al*(70) found telomerase activity in 73% of patients with stage IIIB and IV non-small cell lung cancer with disseminated tumor cells, and 72% of patients with Dukes’ stage C and D colon cancer. On the other hand, Soria *et al*(71) found telomerase activity in the peripheral blood of 21 of 25 patients with stage IV breast cancer. In both studies, telomerase activity was not detected in cells from healthy volunteers. Thus, detection of telomerase activity in the blood or bone marrow appears to be highly suggestive of disseminated cancer cells.

Pfitzenmaier *et al*(72) focused on telomerase activity in disseminated tumor cells in the peripheral blood or bone marrow. The objective of this study was to isolate homogeneous pools of disseminated epithelial cells from bone marrow specimens of patients with clinically localized prostate cancer obtained before radical prostatectomy (RP). These cells were analyzed for telomerase activity, and associations with clinical variables were investigated. This study shows the feasibility of isolating disseminated cancer cells for analyzing individual or pooled cells. Compared to tissue staining, where telomerase is detected in 80–90% of samples, they found lower rates of telomerase activity in the disseminated tumor cells (49%). Telomerase-negative cells might provide information on cell dormancy, as telomerase is a marker of cell proliferation in immortal and cancer cells. Telomerase-positive cells might predict early disease recurrence, but a longer follow-up is needed to test this possibility.

In general, it has been accepted that telomeric shortening is responsible of limiting the half-life of normal human fibroblasts, as well as the expression of telomerase in the cells is sufficient to overcome both replicative senescence and crisis and to give them immortality. Although, the mechanisms involved in telomerase regulation have not been completely solved, the progressive understanding of them is creating the basis needed for research and manipulation of telomerase activity as a potential therapeutic target against cancer.

## 5. Other diseases associated with telomerase activity

The first disease-associated with mutations in human telomerase were identified in patients afflicted with a rare, multi-system disorder called dyskeratosis congenital (73). The clinical manifestations of dyskeratosis congenital generally appear during childhood and include a monocutaneous triad of abnormal skin pigmentation, nail dystrophy and oral leukoplasia. The symptoms are accompanied by a spectrum of other somatic abnormalities such as developmental delay, premature hair loss and organ failure. Bone marrow failure is the principal cause of premature mortality. More recently, telomerase mutations have been detected in the context of aplastic anemia (74), Hoyeraal-Hreidarsson syndrome (75) and idiopaty pulmonary fibrosis (76). Aplastic anemia is a hematological disorder characterized by reduced red blood cell counts, bone marrow failure and liver and lung disease. Hoyeraal-Hreidarsson syndrome is a multisystem disorder characterized by bone marrow failure, immunodeficiency and severe growth retardation. Idiopathic pulmonary fibrosis is a chronic, progressive, and fatal disease that is defined by irreversible lung fibrosis. The unifying molecular characteristic of these diseases is that patients harbor telomeres that are significantly shorter than age-matched control subjects (77).

## 6. Telomerase: structure and function

**Figure 4.**
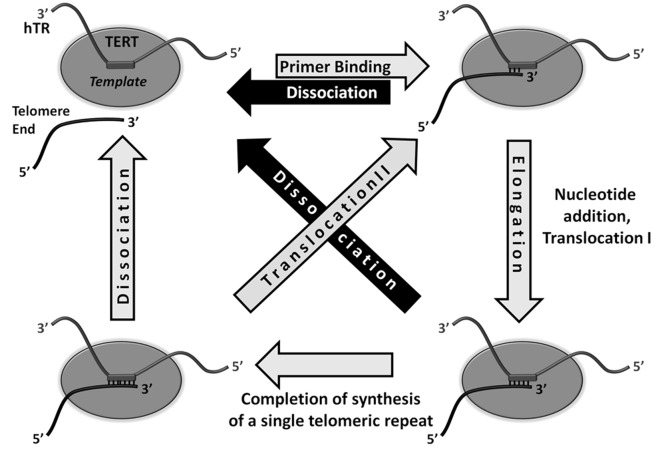
Schematic representation of the synthesis of telomere by telomerase.

**Figure 5.**
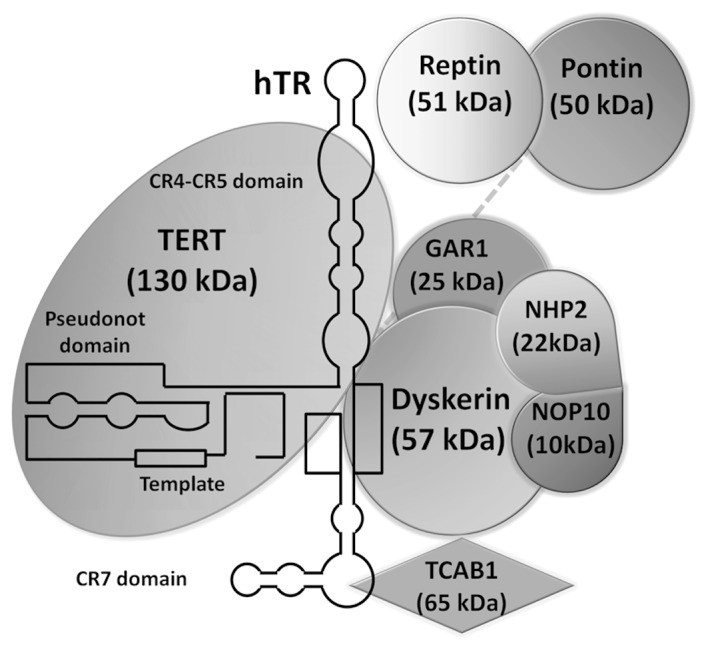
Schematic representation of telomerase and its associated proteins.

In most mammals, the maintenance of telomeric length is carried out mainly by a specific reverse transcriptase, called telomerase that was initially identified in ciliates (6). The human holoenzyme telomerase is a ribonucleoprotein composed by a catalytic subunit, hTERT and an RNA component (hRT) which acts as a template for the addition of a short repetitive sequence dTTAGGG)n in the 3′ end of the telomeric DNA and species-specific accessory proteins (Fig. 4) (12). These accessory proteins regulate telomerase biogenesis, subcellular localization and function *in vivo*. For instance, analysis of affinity-purified telomerase from HeLa cells has identified integral protein components of human telomerase: dyskerin, NHP2; NOP10, pontin/reptin, Gar1 and TCAB1 (78) (Fig. 5). Dyskerin, NHP2 and NOP10 are required for the stability and accumulation of human telomerase RNA (hTR) *in vivo*(78). The heterotrimer of dyskerin, NOP10, and NHP2 is deposited onto each hairpin unit of the H/ACA motif in a highly chaperoned biogenesis process. Cotranscriptional association of the heterotrimer is followed by an exchange of biogenesis factors for the fourth-core subunit, GAR1, to produce a biologically functional RNP. Pontin and reptin are two closed ATPases necessary for the stability of dyskerin and hTR *in vivo*(79). The current model is that dyskerin, pontin and reptin form a scaffold that recruits and stabilizes hTR, and assembles the telomerase ribonucleoprotein particle. Once this complex is formed, pontin and reptin are believed to dissociate from the complex and release the catalytically active enzyme (79). The subcellular location of telomerase appears to be regulated by the recently identified TCAB1 (80). Also, while one study claims that the human telomerase holoenzyme contains only dyskerin, TERT, and hTR (81), other studies establish that the human telomerase holoenzyme assembles all of the core proteins (78).

Besides its association with telomerase, dyskerin is a highly conserved nucleolar protein that, as part of a specialized nucleolar RNP, catalyses the pseudouridylation of specific residues in newly synthesized ribosomal RNAs and spliceosomal snRNA. Pontin and reptin have multiple roles. Both ATPases are associated with several chromating remodeling complexes and have many functions including transcriptional regulation, DNA damage repair and telomerase activity. They can also interact with major oncogenic actors such as β-catenin and c-myc and regulate their oncogenic function. Further studies are needed to elucidate the biochemical and molecular significance of the intricate network of protein-protein and protein-nucleic acid interactions within the telomerase holoenzyme. Moreover, it will be important to investigate whether the composition of the holoenzyme changes in specific stages of the cell cycle. Normal human diploid cells that express hTERT in transient form, acquires telomerase activity, demonstrating that hTERT is the limiting component needed for the restoration of the telomerase activity in these cells (64). The gen hTERT, of ∼37 kb, is present in the human genome as a single copy sequence in the chromosome 5p15.33 (82). hTERT is a relatively large protein (127 kDa) and the reverse transcriptase motifs are found in its C-terminal end. Both, bioinformatics and mutational studies have collectively established that TERT contains three main structural elements: i) a long N-terminal extension that contains conserved DNA and RNA-binding domains, ii) a central catalytic RT domain and iii) a short C-terminal extension. The N-terminal extension of most TERTs contains two conserved domains, the TEN domains and telomerase RNA-binding domain (TRBD). The catalytic domain of TERT is the most characterized region of the protein and contains seven evolutionarily conserved RT motifs, essential for enzymatic activity (83). In contrast to the N-terminus and RT domain, the C-terminus of TERT shows only weak sequence conservation suggesting that it may have species-specific functions or that different amino acid sequences have evolved to fold into similar structural domains.

The RNA molecule of telomerase has been isolated from ciliates, yeasts, and mammals and is essential for telomerase activity (84), but is not a limitating factor for telomerase (85). In vertebrates, RNA of telomerase has an extension between 382 and 559 nucleotides (86), and in humans in particular the RNA component has an extension of 451 nucleotides of length and contains a sequence of 11 bp (5-CUAACCCUAAC-3′) that encodes for telomeric repeats (87). The gen hTR is present in the human genome also as a single copy in chromosome 3q26.

Phylogenetic comparative analysis of vertebrate TR predicts three conserved domains: i) the pseudoknot/template core domain, ii) the CR4/CR5 domain and iii) a box H/ACA domain (88). The core domain is essential for telomerase activity *in vitro* and *in vivo*. The CR4/CR5 is required for telomerase activity but is not essential for mTR stability in the cell and the box H/ACA domain is essential for TR stability, processing, nuclear localization and telomerase activity *in vivo*(89).

Elongation of the telomere by telomerase is a process that happens in different stages. First, the nucleotides of the 3′ extreme of the telomeric DNA are hybridized to the end of the RNA template, inside the RNA domain of the telomerase complex. The template sequence of 11 nucleotides is complementary to almost two telomeric repeats. Secondly, the gap in the extreme of the template is completed by synthesis, using triphosphate nucleotides in the catalytic site of the enzyme (hTERT). In this way, a complete hexanucleotidic repeat is assembled in the template. Finally, the synthesized strand is translocated in 5′ direction in order to allow the formation of a new gap and the repetition of the cycle.

## 7. Other telomerase functions

Telomerase is essential for the long-term proliferation potential of stem cells and cancer cells, and for normal tissue renewal. However, other functions have been described beyond its action at the telomeric level. Indeed, TERT can function as a transcriptional modulator of the Wnt-β-catenin signaling pathway (90). TERT functions as a cofactor in a β-catenin transcriptional complex through interactions with BRG1, which is an SWI/SNF related chromatin remodeling protein. In addition, TERT in a complex with RMRP can act as an RNA-dependent RNA polymerase (91). The TERT-RMRP complex acts as an RDRP and processes RMRP into double-stranded RNA (dsRNA), which is then processed by the endoribonuclease Dicer into small interfering RNA (siRNA), which controls RMRP endogenous levels. Thus, TERT-RMRP-RDRP regulates RMRP levels by a negative-feedback control mechanism. Some evidence has been found for a role for telomerase in the regulation of apoptosis in a telomere maintenance-independent manner (92). TERT contains a mitochondrial localization signal peptide at its N-terminal that targets TERT to mitochondria where it is active (93). Furthermore, it was shown that telomerase sensitizes mitochondrial DNA to hydrogen peroxide-induced oxidative damage, probably through the modulation of metal homeostasis (93). The mitochondrial localization of telomerase also has an important role in apoptosis (94).

## 8. Conclusions

Although pharmacological strategies for affecting telomerase activity are beyond the scope of this review, an excellent review by Tárkányi and Aradi (95) has been published. Although telomerase therapeutics are not approved yet for clinical use, we can assume that based on the promising *in vitro* and *in vivo* results and successful clinical trials, it can be predicted that telomerase therapeutics will be utilized soon to combat malignancies and degenerative diseases. The active search for modulators is justified, because telomere/telomerase system is an extremely promising target offering possibilities to decrease the viability of the cell for therapeutic purposes. The knowledge of this system is vital to fulfill that aim.
